# Increased risk of malignancy in patients with an aortic aneurysm: a nationwide population-based retrospective study

**DOI:** 10.18632/oncotarget.20181

**Published:** 2017-08-11

**Authors:** Jen-Chun Wang, Wu-Chien Chien, Chi-Hsiang Chung, Wen-I Liao, Chang-Huei Tsao, Yung-Fu Wu, Shih-Hung Tsai

**Affiliations:** ^1^ Department of Emergency Medicine, Tri-Service General Hospital, National Defense Medical Center, Taipei, Taiwan; ^2^ Institute of Clinical Medicine, National Yang-Ming University, Taipei, Taiwan; ^3^ Department of Medical Research, Tri-Service General Hospital, National Defense Medical Center, Taipei, Taiwan; ^4^ School of Public Health, National Defense Medical Center, Taipei, Taiwan; ^5^ Taiwanese Injury Prevention and Safety Promotion Association, Taipei, Taiwan; ^6^ Department of Microbiology and Immunology, National Defense Medical Center, Taipei, Taiwan

**Keywords:** aortic aneurysm, malignancy, transforming growth factor-β, Marfan syndrome, fibrillin

## Abstract

**Background:**

Cardiovascular disease and malignancy have numerous similarities and possible interactions, as these diseases share several risk factors, epidemiological features and biological signaling pathways. Data regarding the risk of malignancy in patients with aortic aneurysm (AA) are scarce. We aimed to determine whether patients with AA have an increased risk of malignancy.

**Materials and Methods:**

The data for the nationwide population-based retrospective cohort study described herein were obtained from the Taiwan National Health Insurance Research Database (NHIRD). We selected adult patients who had been newly diagnosed with AA and were followed up between 2000 and 2010. We excluded patients who had been diagnosed with AA and malignancy prior to the date of the AA diagnosis. The control cohort was selected from individuals who had no history of AA and was selected with 1:4 matching according to co-morbidities and medication history. The outcome was a diagnosis of malignancy and the cumulative incidence of AA.

**Results:**

A total of 10,933 patients diagnosed with AA were identified. The patients with an AA had a significantly higher cumulative risk of developing malignancies in subsequent years than the patients without an AA (log rank test < 0.001). Similarly, patients with malignancies had a significantly higher cumulative risk of developing an AA in subsequent years than patients without malignancies (log rank test < 0.001).

**Conclusions:**

Patients with an AA were shown to have a substantially increased risk of developing a variety of malignancies compared with patients without AAs. Healthcare professionals should be aware of this increased risk when treating patients with AAs.

## INTRODUCTION

Aortic aneurysms(AAs) are a common cause of sudden death. Most AAs are discovered accidentally, but progressive aneurysmal enlargement can lead to rupture. As a result of increased surveillance and surgical innovations, the rate of AA-related mortality has decreased drastically [1]. Previous small-scale clinical observational studies have shown that cardiovascular disease (CVD) events were the most frequent cause of death after endovascular surgery, whereas malignancy-related death accounted for 14.5–29% of deaths after endovascular surgery, following CVD- and AA-related deaths (35%) [2]. Although commonly considered two separate disease entities, CVD and malignancy have numerous similarities and possible interactions, as they share several risk factors, epidemiological features and biological signaling pathways [3].

Advances in molecular biological techniques have led to the discovery of increasing evidence suggesting that several related biological signaling pathways are associated with the development of malignancies and AAs. Recent studies have shown that the transforming growth factor-β (TGF-β), mitogen-activated protein kinase (MAPK),and hypoxia inducible factor-1α (HIF-1α) pathways in addition to inflammatory pathways regulate malignant cell initiation, proliferation, migration and invasion [4–6] and that these pathways are also active participants in the pathogenesis of AAs.

Specifically, TGF-β signaling overactivation is associated with Marfan syndrome (MFS) and several MFS-like conditions. Those diseases are also clearly associated with non-inflammatory structural degenerative CVDs, including aortic root dilatation, thoracic aneurysms and aortic dissection [7, 8]. In tumor cells, TGF-β loses its anti-proliferative characteristics and becomes an oncogenic factor, which leads to impairments in its signaling pathway in various solid tumors and hematological malignancies [9].

Studies regarding the associations between AAs and subsequent or concurrent malignancies are scarce. Thus, we aimed to determine whether patients with aortic diseases have an increased risk of malignancy using a nationwide health care insurance claim database.

## RESULTS

**Figure 1:**
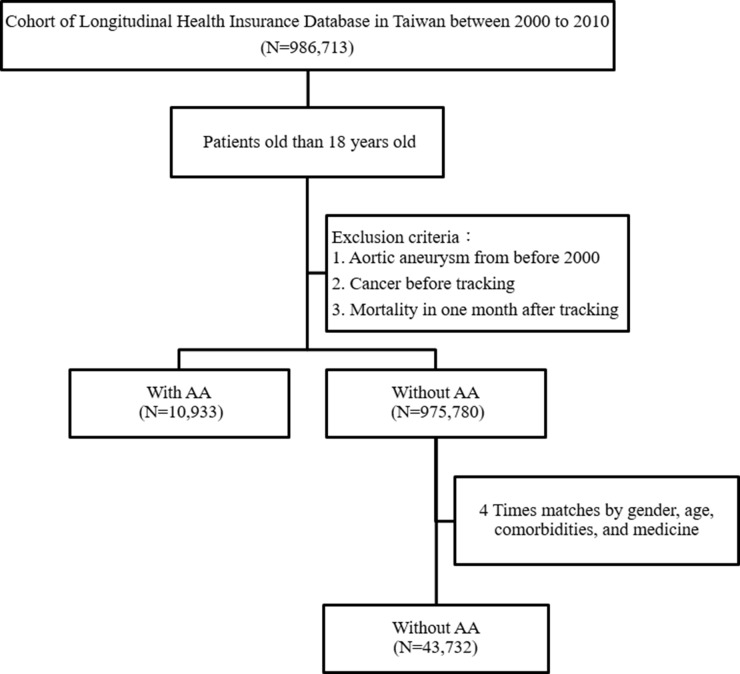
Patient selection flowchart AA = aortic aneurysm

A flow diagram of our patient enrollment scheme is presented in Figure 1. A total of 10,933 patients diagnosed with AA were identified in the National Health Insurance Research Database (NHIRD), which contains a total of 986,713 patients. Another 43,732 age-, gender-, and comorbidity-matched patients were designated controls. As shown in Table 1, as expected, there were no significant differences in gender, age and co-morbidities, including hypertension, hyperlipidemia, diabetes mellitus (DM) and chronic obstructive pulmonary disease (COPD), between the two groups after matching. There were also no significance differences between the two groups with regard to medication use. Table 2 shows the incidences of malignancies during the ten-year follow-up period. At the end of the follow-up period, patients with an AA had significantly higher incidences of hypertension (54.60% vs. 31.17%, *p* = 0.001) and hyperlipidemia (3.54% vs. 2/65%, *p* < 0.001) but lower incidences of DM (10.68% vs. 15.93%, *p* < 0.001) and COPD (7.28% vs. 13.21%, *p* < 0.001) than patients without an AA.

**Table 1:** Baseline characteristics of patients included in the study

	Patients with AA N = 10,933	Patients without AA N = 43,732	*p* value
Gender			0.999
Male	7,783 (71.19%)	31,132 (71.19%)	
Female	3,150 (28.81%)	12,600 (28.81%)	
Age (years)	63.25±14.30	65.50±15.78	0.121
Age groups (years)			0.386
20–49	2.745 (25.11%)	9,127 (20.87%)	
50–65	1,660 (15.18%)	8,560 (19.57%)	
> 65	6,528 (59.71%)	26,045 (59.56%)	
Comorbidities			
Hypertension	6,522 (59.65%)	26,088 (59.65%)	0.999
Diabetes	992 (9.07%)	3,968 (9.07%)	0.999
Hyperlipidemia	400 (3.66%)	1,600 (3.66%)	0.999
COPD	663 (6.06%)	2,652 (6.06%)	0.999
Medicine			
β-blockers	2,721 (24.89%)	11,167 (25.54%)	0.462
CCBs	2,820 (25.79%)	11,611 (26.55%)	0.338
ACEIs	2,632 (24.07%)	10,637 (24.32%)	0.296
ARBs	2,391 (21.87%)	9,580 (21.91%)	0.475
Diuretics	2,290 (20.95%)	8,967 (20.50%)	0.556
Statins	2,484 (22.72%)	9,725 (22.24%)	0.279

COPD = chronic obstructive pulmonary disease; CCBs = calcium channel blockers; ACEIs = angiotensin-converting-enzyme inhibitors; ARBs = angiotensin receptor blockers.

**Table 2:** Factors of malignancy with catastrophic illness at the end of follow-up stratified according to the variables listed in the table based on Cox regression

	Patients with AA			Patients without AA			Ratio	Adjusted HR^*^	95% CI	p value
	Events	PY	Incidence rate (per 105 PY)	Events	PY	Incidence rate (per 105 PY)				
Total	396	15,140.57	2,615.49	1,726	85,732.47	2,013.24	1.299	2.753	2.452–3.090	< 0.001
Age										
20–49	19	1,533.39	1,239.08	90	8,086.30	1,112.99	1.113	2.256	1.975–2.552	< 0.001
50–65	87	4,231.04	2,056.23	221	12,280.43	1,799.61	1.143	2.458	2.110–2.715	< 0.001
> 65	290	9,374.14	3,093.62	1,415	64,645.74	2,188.85	1.413	2.976	2.445–3.469	< 0.001
Gender										
Male	293	10892.31	2,689.97	1,311	62,108.09	2,110.84	1.274	2.697	2.358–3.085	< 0.001
Female	103	4,248.46	2,424.41	415	23624.38	1,756.66	1.380	2.926	2.329–3.675	< 0.001
Comorbidities†										
With	206	5,855.79	3,517.89	1,017	39,465.57	2,576.93	1.365	2.769	2.373–3.232	< 0.001
Without	190	9,284.78	2,046.36	709	46,266.90	1,532.41	1.335	2.975	2.507–3.530	< 0.001

*p* values are for the adjusted HRs. PY = person-years. HR = hazard ratio. Events = number of cancer diagnoses. ^*^Adjusted HR was adjusted for age, sex, and comorbidities. †Comorbidities included hypertension, diabetes, hyperlipidemia and chronic obstructive pulmonary disease.

**Figure 2:**
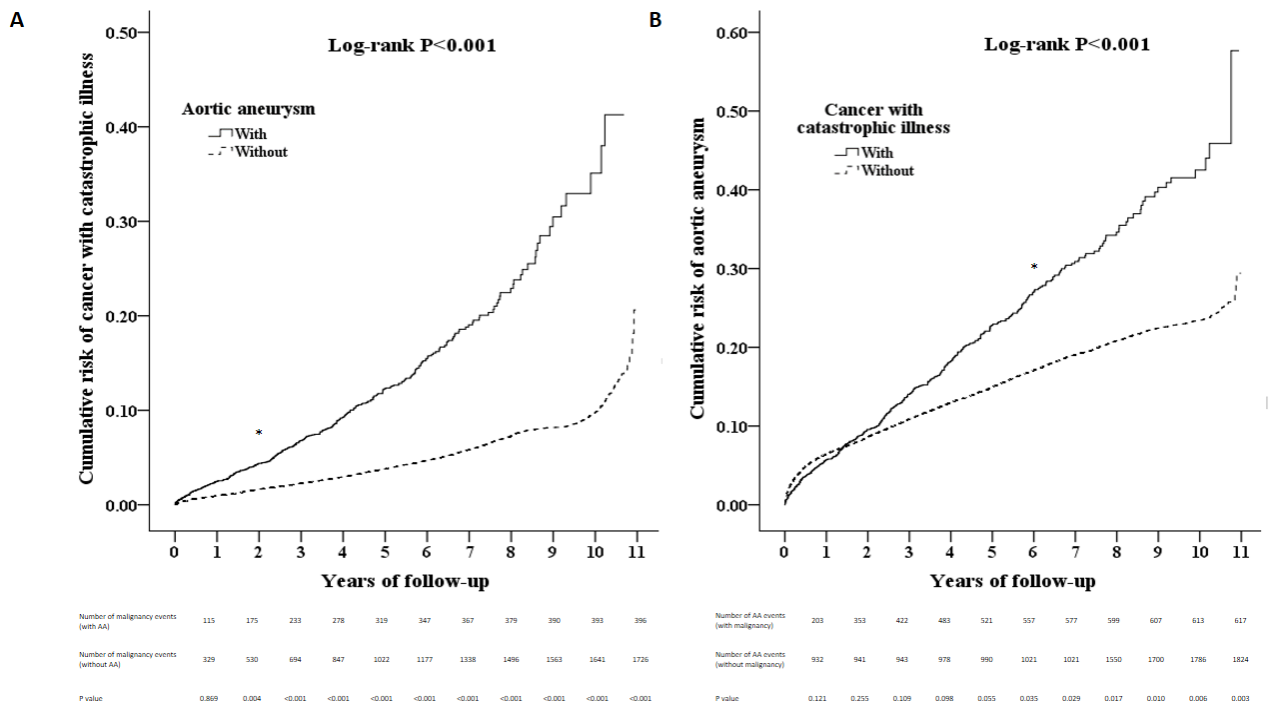
(**A**) The Kaplan-Meier curve for the cumulative risk of malignancy events by AA (log rank *p* < 0.001) (**B**). The Kaplan-Meier curve for the cumulative risk of AA events by malignancy (log rank *p* < 0.001) AA = aortic aneurysm.

Patients with an AA had a significantly higher cumulative risk of developing malignancies in subsequent years than patients without an AA (log rank test < 0.001, Figure 2A). Similarly, patients with malignancies had a significantly higher cumulative risk of developing an AA in subsequent years than patients without malignancies(log rank test < 0.001, Figure 2B). Patients with an AA had a significantly higher risk of developing malignancies than patients without an AA, independent of the effects of gender, age and co-morbidities (Table 2).

As shown in Table 3, AAs were associated with both hematological malignancies and solid malignancies. Among solid malignancies, head and neck, liver, pancreas, lung, skin, breast, cervix, prostate, bladder, and kidney malignancies were significantly associated with AAs. The remaining malignancies, which were not significantly associated with AAs, are shown in Supplementary Table 1.

**Table 3:** Comparison of individual malignancy risk in the AA cohort and the control cohort

	Patients with AA		Patients without AA		Ratio	Adjusted HR^*^	95% CI	p value
	Events	Incidence rate	Events	Incidence rate				
Head and neck	29	191.54	116	135.30	1.416	1.514	1.016–2.300	0.025
Colon	38	250.98	161	187.79	1.336	3.394	2.326–4.952	< 0.001
Rectum	26	171.72	97	113.14	1.518	2.805	1.454–1.767	< 0.001
Liver and intrahepatic bile ducts	64	422.71	312	363.92	1.162	2.588	1.953–3.431	< 0.001
Pancreas	10	66.05	47	54.82	1.205	2.696	1.313–5.536	0.007
Retroperitoneum and peritoneum	4	26.42	3	3.50	7.550	16.124	3.411–76.223	< 0.001
Trachea, bronchus and lung	62	409.50	257	299.77	1.366	3.278	2.444–4.396	< 0.001
Skin	7	46.23	30	34.99	1.321	2.067	1.079–4.862	0.026
Female breast	8	52.84	38	44.32	1.192	2.812	1.272–6.220	0.011
Uterus	2	13.21	1	1.17	11.325	21.507	1.725–268.192	0.017
Cervix	7	46.23	27	31.49	1.468	2.487	1.035–5.971	0.042
Prostate	28	184.93	100	116.64	1.585	4.897	3.135–7.650	< 0.001
Bladder	33	217.96	81	94.48	2.307	5.206	3.386–8.005	< 0.001
Kidney and other urinary organs	21	138.70	68	79.32	1.749	3.173	1.889–5.329	< 0.001
Hematopoietic malignancy†	27	178.33	139	162.13	1.100	2.464	1.593–3.810	< 0.001

^*^Adjusted hazard ratio was adjusted for age, sex and comorbidities. †Hematopoietic malignancy including leukemia and lymphoma.

## DISCUSSION

The major finding of the study was that patients with an AA have an increased risk of developing both solid and hematological malignancies in subsequent years, even after adjustment for certain co-morbidities, including hypertension, DM, hyperlipidemia and COPD. Patients with malignancies were also prone to developing AAs.

Previous clinical observational studies have shown that an association exists between AAs and increased incidence of malignancy. Serial CT surveillance revealed that the rate of pulmonary malignancies was high in a population with AAs compared with a population without AAs [10]. This finding raised the question of whether increased medical surveillance for AAs may provide clinicians with opportunities diagnose malignancies earlier. Age, male gender, hyperlipidemia, hypertension, obesity and tobacco use are well known risk factors for AA [11]. Several modifiable CVD risk factors are shared between AA and malignancies, including tobacco use, hypertension, obesity and hyperlipidemia [3]. In this study, we found that the patients with an AA had an increased risk of developing both solid and hematological malignancies in subsequent years and vice versa. We hypothesized that these shared risk factors could also contribute to the concurrency of AA and malignancies. Patients with AAs were found to be significantly more likely to develop malignancies than patients without AAs 2 years after their diagnoses of AA, and patients with malignancies were found to be significantly more likely to develop AAs than patients without malignancies 5 years after their diagnoses of malignancy. Based on these findings, we hypothesized that the concurrence of AAs and malignancies may be attributed to risk factors shared by the two diseases, as well as to changes in the activity of biological signaling pathways common to both disease processes. The results of regression analyses performed in previous studies have suggested that age and tobacco use rather than AAs are independent risk factors for the development of cancer [12]. We found that patients with AAs still had a higher risk of developing malignancies than patients without AAs, even after adjustment for age, gender and several co-morbidities. We are aware that certain medications, such as statins and renin–angiotensin system inhibitors, have been shown to have protective effects in the development of experimental AA. Nonetheless, there is no evidence that commonly used cardiovascular drugs have beneficial clinical effects on AA progression [13]. The current evidence is not sufficient to support using aspirin as an adjuvant therapy for colorectal cancer [14–16]. After matching for co-morbidities, we found that there were no differences regarding those medications. Therefore, the biological signaling pathways that are involved in both AAs and malignancies should be explored to elucidate this relationship further.

Matrix metalloproteinases (MMPs) are proteolytic enzymes, and MMPs expression and activation are regulated in physiological conditions to prevent uncontrolled body tissue destruction. Degradation of the collagen and elastin of the aortic wall is induced by altering of the expression of several catabolic MMPs [17]. Patients with AAs showed significantly higher MMPs expression levels in their aortic tissue than patients without AAs [18, 19]. MMPs are known as regulators of the tumor microenvironment, and numerous studies have confirmed the existence of associations between MMPs and tumor growth, tumor apoptosis, tumor vasculature, and neoplastic progression, invasion and metastasis initiation in malignancies [20, 21].

Recent studies have provided convincing evidence indicating that several signaling pathways are involved in both AAs and malignancies, including the TGF-β, MAPK JNK, ERK, Rho/ROCK, NF-kb, HIF-1α, and MMPs signaling pathways. The MAPK JNK and ERK pathways are cancer cell proliferation, differentiation, survival, inflammation and drug resistance pathways [22, 23], the suppression of which has been shown to reduce the growth of AAs in experimental AA models [24, 25]. HIF-1α signaling is associated with tumor metastasis, tumor angiogenesis, and a poor patient prognosis, as well as resistance to tumor therapy [26]. HIF-1α overexpression has been found at the edges of ruptured human AA tissue [27]. In vascular cells, HIF-1α promotes vascular inflammation, MMPs release and vascular remodeling [28, 29]. HIF-1α inhibition has been shown to attenuate AA progression through MMP down-regulation [30]. Small molecules developed for anti-cancer purposes have also been shown to be effective in the treatment of experimental AA [30–32].

TGF-β signaling plays critical roles in the pathogenesis of AAs [33]. Cardiovascular abnormalities are the major cause of morbidity and mortality in MFS and several clinically related diseases, including Loeys-Dietz syndrome, Shprintzen-Goldberg syndrome, aneurysm-osteoarthritis syndrome and thoracic aortic aneurysm syndrome, in which the pathogeneses are driven by dysregulated TGF-β signaling [8]. MFS is characterized by a deficiency of fibrillin-1 (FBN1) in the extracellular matrix. Studies of Marfan murine models have revealed that the TGF-β signaling pathway is heavily involved in the pathogenesis of MFS. In a Korean MFS cohort, up to 89.8% of patients who had FBN1 mutationsin genetic analysis developed aortic root aneurysm/dissection, and 62% of patients underwent some type of cardiovascular surgery. CVD manifestations were commonly detected, and FBN1 mutations were present in approximately 90% of patients [34]. AAs in MFS tended to occur in relatively young patients and are susceptible to dissection and rupture [35, 36]. Targeting the over activity of the TGF-β pathway has been proposed as a strategy for treating AAs [37]. TGF-β-induced epithelial-mesenchymal transition and reversion from the mesenchymal to the epithelial phenotype have been increasingly recognized as integral aspects of cancer progression that contribute to malignant cell survival and dissemination [38]. TGF-β actively participates in the pathogenesis of several malignancies [39–41]. Notably, accumulated case reports have indicated that an association exists between MFS/MFS-like conditions and several hematological and solid malignancies [42]. However, the associations between MFS/MFS-like conditions and malignancies have not been evaluated in large-scale studies.

### Limitations

The strength of our study was its population-based cohort design. We determined the incidence of cancer by recording both ICD-9 codes and data from the Registry of Catastrophic Illness Patient Database to increase the accuracy of our results. We excluded cofounding factors, including co-morbidities and medication effects, from our analysis. However, there were some limitations to this study. Although we aimed to control for potential disease-associated confounders to the greatest extent possible, unknown or unmeasured confounders may have biased our findings. The NHIRD registry is not able to provide detailed information regarding the laboratory results or health-related lifestyle factors of patients, such as alcohol consumption, tobacco use, body mass indices and family histories, all of which can increase the risk of cancer and were potential confounding factors in this study; however, we considered the incidence of COPD a proxy variable for tobacco use to attenuate its potential confounding effect [43]. Both mechanistic studies and animal experiments are required to further clarify this important issue.

## MATERIALS AND METHODS

### Data source

The data for the nationwide population-based retrospective cohort study described here in were obtained from the Longitudinal Health Insurance Database 2005 (LHID 2005), a subset database of the Taiwan NHIRD. This National Health Insurance program was implemented in 1995 and provides health care coverage for 99% of the population in Taiwan (more than 23 million people). The LHID 2005 contains information on medical service utilization by a randomly selected sample of approximately one million beneficiaries, who represent approximately 5% of Taiwan's population, in 2005. The information was extracted from the NHIRD between 2000 and 2010. The accuracy of the diagnoses in the NHIRD, namely, the diagnoses of major diseases such as stroke and acute coronary syndrome, has been validated [44, 45]. The LHID 2005 consists of “de-identified” secondary data released to the public for research purposes. Patient identification numbers, genders, birthdays, dates of admission and discharge, ICD-9-CM (International Classification of Diseases, 9th Revision, Clinical Modification) diagnostic and procedure codes (up to five each), and outcomes are encrypted, as is information regarding the medical institutions at which patients received their services. This study was conducted in accordance with the Declaration of Helsinki and relevant guidelines and was approved by the Institutional Review Board of Tri-Service General Hospital, National Defense Medical Center, Taipei, Taiwan (TSGH IRB No.B-104-21).

### Sampled patients

This study featured study and comparison cohorts. Using the LHID 2005, we selected adult patients aged >18 years who had been newly diagnosed with AAs (ICD-9-CM 441.1–441.9) and were followed up between 2000 and 2010. We excluded patients who had been diagnosed with AAs (ICD-9-CM 441.1–441.9) and cancer (recorded from the Registry of Catastrophic Illness Patient Database) prior to the index date. Patients who suffered mortality within one month after tracking initiation were also excluded from the study. The date of AA diagnosis was used as the index date. The control candidate sampling comparisons were selected from individuals in the LHID 2005 who had no history of AA. The patients and control cohorts were selected by 1:4 matching, according to the following baseline variables: age; sex; co-morbidities, including hypertension (ICD-9-CM 401–405), DM(ICD-9-CM 250), hyperlipidemia (ICD-9-CM 272.0–272.4), and COPD(ICD-9-CM 490–496); and medication history, including β-blocker, calcium channel blocker, angiotensin-converting enzyme inhibitor, angiotensin receptor blocker, and diuretic history. The index dates for control patients were the same as the corresponding dates for patients with AAs. A similar approach was used for malignancy, as patients with malignancy were identified as the study group, and patients without malignancy were identified as the control group by1:4 matching, according to baseline variables, including age, sex, co-morbidities and medication history.

### Outcome measurements

The study outcome was a diagnosis of malignancy during the 10-year follow-up period. Malignancy events were identified from the Registry of Catastrophic Illness Patient Database, a subset of the NHIRD. Patients newly diagnosed with cancer can apply for a catastrophic illness certificate issued by the government. Before issuing a catastrophic illness certificate, a review of medical records, imaging results, and pathology reports is performed by a panel of specialists and experts on the specific disease. The use of both the NHIRD and catastrophic illness certificate to identify the development of malignancies has been well-validated internally and externally in several studies [44, 46, 47]. ICD-9 codes pertaining to malignancies involving different areas of the body, including malignancies of the head and neck (ICD-9-CM codes 140–149); esophagus (ICD-9-CM 150); stomach (ICD-9-CM 151); small intestine (ICD-9-CM 152); colon and rectum (ICD-9-CM 153–154); liver (ICD-9-CM 155); gallbladder and extrahepatic bile ducts (ICD-9-CM 156); pancreas (ICD-9-CM 157); retroperitoneum and peritoneum (ICD-9-CM 158); nasal cavities, middle ear and accessory sinuses (ICD-9-CM 160); larynx (ICD-9-CM 161); lung (ICD-9-CM 162); pleura (ICD-9-CM 163); thymus, heart and mediastinum (ICD-9-CM 164); bone and articular cartilage (ICD-9-CM 170); connective and other soft tissues (ICD-9-CM 171); skin (ICD-9-CM 172–173); female breast (ICD-9-CM 174); uterus (ICD-9-CM 179); cervix (ICD-9-CM 180); body of the uterus (ICD-9-CM 182); ovary and other uterine adnexa (ICD-9-CM 183); prostate (ICD-9-CM 185); penis and other male genital organs (ICD-9-CM 187); bladder (ICD-9-CM 188); kidney (ICD-9-CM 189); brain (ICD-9-CM 191); thyroid (ICD-9-CM 193); other endocrine glands and related structures (ICD-9-CM 194); and blood (ICD-9-CM 200–208). Another outcome assessed in this study was the cumulative incidence of AA, which was compared between malignancy and non-malignancy patients, during the 10-year follow-up period.

### Statistical analysis

The clinical characteristics of the patients enrolled in the study are expressed in numerical form. Categorical variables, which a presented as percentages, were compared using chi-squared tests and Fisher's exact test. Continuous variables, which are presented as the mean and standard deviation, were compared using *t-tests*. The primary goal of the study was to determine whether patient clinical characteristics were associated with malignancies. The associations between time-to-event outcomes (prognoses) and clinical characteristics were investigated using Kaplan-Meier survival analysis and multivariate Cox regression analysis with forward stepwise selection. The results are presented as adjusted hazard ratios (HRs) with corresponding 95% confidence intervals (CIs). The threshold for statistical significance was *p* < 0.05. All data analyses were conducted using SPSS software, version 18 (SPSS Inc., Chicago, IL, USA).

## CONCLUSIONS

Patients with AAs were shown to have a substantially increased risk of developing a variety of malignancies compared with patients without AAs. Thus, increased cancer surveillance may be needed in these patients. Health care professionals should be aware of this increased risk when treating patients with AAs.

## SUPPLEMENTARY MATERIALS TABLE



## Abbreviations

CIs: confidence intervals
COPD: chronic obstructive pulmonary disease
DM: diabetes mellitus
FBN-1: fibrillin-1
HR: hazard ratio
ICD-9-CM: International Classification of Diseases, 9th Revision, Clinical Modification
LHID: Longitudinal Health Insurance Database
MFS: Marfan syndrome
NHIRD: National Health Insurance Research Database
ORs: odds ratios
TGF-β: transforming growth factor-β.
