# Supplementary material for: Increased risk of malignancy in patients with an aortic aneurysm: a nationwide population-based retrospective study
Source: Oncotarget. 2017 Aug 11;9(2):2829–37. doi: 10.18632/oncotarget.20181 (PMC5788683; doi:10.18632/oncotarget.20181)
Supplement: Supplementary file 1 [file oncotarget-09-2829-s001.pdf]

Increased risk of malignancy in patients with an aortic aneurysm: a nationwide population-based retrospective study

SUPPLEMENTARY MATERIALS

Supplementary Table 1: Comparison of individual cancer risk in the AA cohort and the control cohort

	Patients with AA		Patients without AA		Ratio	Adjusted HR*	95% CI	*P* value
	Events	Incident rate	Events	Incident rate				
Esophagus	5	33.02	48	55.99	0.590	0.994	0.400–2.727	0.830
Stomach	23	151.91	131	152.80	0.994	0.986	0.254–2.775	0.465
Small intestine	2	13.21	8	9.33	1.416	2.892	0.550–15.227	0.210
Gallbladder & extrahepatic bile ducts	6	39.63	34	39.66	0.999	1.845	0.737–4.620	0.191
Nasal cavities, middle ear and accessory sinuses	0	0.00	1	1.17	0.000	0.000	-	0.976
Larynx	1	6.60	18	21.00	0.315	0.629	0.081–4.886	0.658
Pleura	0	0.00	6	7.00	0.000	0.000	-	0.971
Thymus, heart and mediastinum	0	0.00	6	7.00	0.000	0.000	-	0.985
Bone and articular cartilage	0	0.00	4	4.67	0.000	0.000	-	0.961
Connective and other soft tissue	1	6.60	5	5.83	1.132	1.939	0.207–18.127	0.562
Kaposi's sarcoma	0	0.00	3	3.50	0.000	0.000	-	0.978
Body of uterus	1	6.60	4	4.67	1.416	3.342	0.321–34.755	0.313
Ovary and other uterine adnexa	1	6.60	4	4.67	1.416	2.583	0.266–25.072	0.413
Penis and other male genital organs	0	0.00	2	2.33	0.000	0.000	-	0.985
Brain	3	19.81	15	17.50	1.132	1.297	0.343–4.901	0.701
Thyroid	2	13.21	11	12.83	1.030	1.652	0.331–8.250	0.541
Other endocrine glands and related structures	1	6.60	2	2.33	2.831	8.271	0.648–105.508	0.104

*Adjusted hazard ratio was adjusted for age, sex and comorbidities. †Hematopoietic malignancy including leukemia and lymphoma.
